# Studies on the Flavonoid Composition of *Pleioblastus amarus* Leaves and Shoots Based on Targeted Metabolomics

**DOI:** 10.3390/metabo15110709

**Published:** 2025-10-30

**Authors:** Yongmei Chen, Qingwen Wei, Jing Wang, Chuanyao Huang, Zhenghao Wang, Yuchun Liu

**Affiliations:** College of Chemical Engineering, Sichuan University of Science & Engineering, Zigong 643000, China; 323086001214@stu.suse.edu.cn (Q.W.); 324086001211@stu.suse.edu.cn (J.W.); 322086001132@stu.suse.edu.cn (C.H.); 322086001122@stu.suse.edu.cn (Z.W.); 323086001205@stu.suse.edu.cn (Y.L.)

**Keywords:** bamboo species, phenolic metabolites, LC–MS/MS quantification, metabolic pathway analysis, tissue-specific differences

## Abstract

**Background:** *Pleioblastus amarus* (Keng) Keng f. is widely distributed in southern China and serves as both a medicinal and edible resource. Flavonoids represent the main bioactive compounds in this species, contributing to its antioxidant, anti-inflammatory, and anti-tumor activities. **Methods:** In this study, a targeted metabolomics approach based on liquid chromatography–tandem mass spectrometry (LC–MS/MS) was applied to analyze 35 flavonoid compounds in dry leaf and shoot samples of *P. amarus*. The method demonstrated a detection limit (LOD) of X μg/g and a quantification limit (LOQ) of Y μg/g, with good linearity within the range of A to B μg/g for all compounds. **Results:** Twenty flavonoids were identified, with cynaroside being the most abundant in leaf samples (0.013 mg/g) and isovitexin being the most abundant in shoot samples (0.001 mg/g). The total flavonoid content in dry leaf (0.19 mg/g) was significantly higher than in dry shoot (0.022 mg/g). Multivariate statistical analyses revealed clear clustering and metabolic differences between the two tissues, and fifteen differential metabolites were identified by orthogonal partial least squares discriminant analysis (OPLS-DA). The correlation and pathway analyses suggested two distinct metabolic groups: one involving catechin, L-epicatechin, formononetin, and glycitin, and another centered on naringenin-derived metabolites. **Conclusions:** These findings provide new insights into tissue-specific flavonoid metabolism in *P. amarus*, highlighting distinct patterns in the leaves and shoots. By comparing flavonoid metabolism in other bamboo species, such as *Phyllostachys edulis* and *Indocalamus*, this study reveals both differences and similarities across species. It enhances our understanding of flavonoid synthesis and distribution in *P. amarus* and supports the development of flavonoid-based pharmaceuticals, offering valuable insights for further biochemical and pharmacological research on bamboo species.

## 1. Introduction

*Pleioblastus amarus* (Keng) Keng f. is a bamboo species belonging to the genus *Pleioblastus* in the subfamily Bambusoideae of the family Poaceae, as described in the Flora of China and other taxonomic monographs [1,2,3]. It is native to and widely distributed in southern China, including Sichuan, Guizhou, and Guangdong provinces [1]. Various parts of *P. amarus* possess high utilization and development value. Its shoots are rich in nutrients and have long been consumed as a green, healthy, organic vegetable [4]. According to traditional Chinese medicine, *P. amarus* also holds medicinal value—its leaves are known to clear heat, improve vision, promote diuresis, and detoxify, making them a natural herbal resource, while the shoots can help eliminate dampness and promote urination [5].

In recent years, numerous studies have confirmed that the leaves and shoots of *P. amarus* are rich in flavonoid bioactive compounds [5,6,7]. Flavonoids are a class of natural polyphenolic compounds with notable physiological activities, including antioxidant, anti-inflammatory, and anticancer properties, as well as roles in enhancing immune function and preventing cardiovascular diseases [8,9]. Flavonoid content and distribution within *P. amarus* exhibit considerable variation, which can be influenced by factors such as plant age, environmental conditions, and tissue type. Liang Qiong et al. [10] isolated apigenin and luteolin from the young stems of *P. amarus*. Using LC-MS/MS technology, Li et al. [11] identified nine flavonoids in *P. amarus* leaves, including quercetin, rutin, luteolin, kaempferol, isovitexin, vitexin, orientin, isoorientin, and tricine. Chu Bingquan et al. [12] systematically analyzed the total flavonoid content in dried leaves from 91 species across 18 genera of Bambusoideae, focusing on key flavonoids such as orientin, isoorientin, vitexin, isovitexin, quercetin, tricin, rutin, and luteolin. Their findings revealed that the average total flavonoid content in *Phyllostachys* leaves was 16.34 mg/g, with isoorientin being the most abundant (0.55 mg/g). *Indocalamus* leaves exhibited a higher average flavonoid content (17.25 mg/g) than *Phyllostachys*, with isoorientin reaching 0.84 mg/g. *Bambusa* leaves showed comparable flavonoid levels (16.27 mg/g) to *Phyllostachys*, but notably higher concentrations of isoorientin (1.39 mg/g) and rutin (1.53 mg/g). In *Pleioblastus* leaves, the average total flavonoid content was lower (12.63 mg/g) than in *Phyllostachys*, with the combined content of four C-glycosylflavonoids being 1.23 mg/g, of which orientin was predominant (0.41 mg/g). Hu Xia et al. [13] reported that the total flavonoid content in *P. amarus* shoots reached 10.655 mg/g, while that in *Phyllostachys nigra* shoots was significantly higher (32.24 mg/g). Yang Yongfeng et al. [5] found that *P. amarus* shoots contained 4.003 mg/g of total flavonoids. To date, research on the types and concentrations of flavonoids in *P. amarus* shoots remains limited, and comparative studies between leaves and shoots are entirely lacking. Furthermore, existing studies have primarily relied on HPLC or LC-MS techniques with specific reference standards for flavonoid analysis in bamboo.

Molecular biology techniques are continuously advancing, and current research on the biosynthetic pathways and regulatory mechanisms of flavonoid compounds is progressively delving into the study of external environmental factors and biological elements [14]. In higher plants, the biosynthesis of flavonoid compounds begins with the stepwise condensation reaction of p-coumaroyl-CoA and three molecules of malonyl-CoA catalyzed by chalcone synthase (CHS) [15]. Subsequently, chalcone isomerase (CHI) mediates the cyclization of chalcone to form naringenin [16]. The flavanone naringenin serves as a key biochemical precursor that can be further derived into various subclasses such as flavonols, anthocyanins, and flavones. The main factors influencing the regulation of flavonoid biosynthesis in plants include environmental and biological elements such as light [17], temperature [18], water [19], salt stress [19], UV radiation [20], as well as regulatory genes [19]. Studies have found that microRNAs (miRNAs), as crucial post-transcriptional regulators, play an important role in fine-tuning flavonoid metabolism by targeting key enzyme genes and transcription factors [21]. Plant hormones also serve a pivotal role in regulating flavonoid metabolism, and it has been proposed that the rational application of plant hormones can enhance the content of flavonoid compounds in plants [22].

Therefore, this study employs targeted metabolomics based on LC-MS/MS technology to comprehensively characterize, for the first time, the major flavonoid compounds present in two distinct parts of *P. amarus* (shoots and leaves), compare their differences, and analyze the metabolic pathways of key flavonoids, thereby laying the foundation for subsequent targeted extraction and isolation of flavonoid compounds from *P. amarus*.

## 2. Materials and Methods

### 2.1. Chemicals and Reagents

LC-MS-grade methanol (>99.9%) was purchased from Thermo (Beijing, China), and LC-MS-grade formic acid (>98%) was purchased from TCI (Shanghai, China). Naringenin was obtained from Sigma-Aldrich (Shanghai, China). Puerarin was obtained from Macklin (Shanghai, China). Chrysin, daidzein, fisetin, kaempferol, luteolin, catechin, quercetin, quercetin 3-glucoside, icariin, naringin, and diosmin were all obtained from Aladdin (Shanghai, China). Genistein and baicalin were obtained from OKA (Beijing, China). Kaempferide, myricetin, silybin, and rutin were all obtained from Shyuanye (Shanghai, China). Biochanin A, epicatechin, and isovitexin were all obtained from Dalian Meilun Biotechnology Co., Ltd. (Dalian, Liaoning Province, China). Liquiritigenin, formononetin, apigenin, glycitein, taxifolin, dihydromyricetin, vitexin, genistin, glycitin, astragalin, quercitrin, cynaroside, and daidzin were all obtained from Chengdu Refensi Biotechnology Co., Ltd. (Chengdu, Sichuan Province, China).

### 2.2. Plant Material and Preparation of Extracts

The leaves and shoots of *P. amarus* were collected in the summer morning of April 2023 in Yibin City, Sichuan Province, China, with 12 samples taken from ten-year-old plants of the same species. Samples 1–12 used in the experiment were cultivated and identified by Yibin Huiming Agricultural Development Co., Ltd. (Yibin, China) (104.76° E, 28.73° N). The specimens were deposited at SUSE (http://www.suse.edu.cn/ accessed on 5 September 2023) with corresponding voucher numbers: KZY001, KZY002, KZY003, KZY004, KZY005, KZY006, KZS001, KZS002, KZS003, KZS004, KZS005, and KZS006. After collection, the leaf and shoot samples were washed and immediately transferred to a bake-out furnace at 60 °C for drying. The dried materials were then ground into a fine powder using a pulverizer (FW-100, Tianfeng, Shanghai, China). One gram of these powdered leaves and shoots was placed into a 2 mL centrifuge tube, and 600 μL of methanol was accurately added and vortexed for 60 s. Two steel balls were added to each tube, and the samples were further ground in a tissue grinder at 60 Hz for 1 min. This grinding step was repeated at least three times. Subsequently, the samples were sonicated at room temperature for 15 min and centrifuged at 12,000 rpm for 5 min at 4 °C. The supernatant was filtered through a 0.22 μm membrane, and the filtrate was added to the LC-MS bottle [23,24]. Three technical replicates were prepared and analyzed for each sample (*n* = 9) to ensure accuracy and precision. The overall results represent two independent experiments (*n* = 18). A quality control (QC) material, consisting of aliquots from all samples, was also prepared to monitor the stability of the samples, the instrumentation, and the analyses.

### 2.3. Liquid Chromatography–Tandem Mass Spectrometry System

Chromatographic separation was performed on an ACQUITY UPLC^®^ BEH C18 column (2.1 × 100 mm, 1.7 μm, Waters, Milford, MA, USA) with an injection volume of 5 μL and column temperature maintained at 40 °C. The mobile phase consisted of 0.1% formic acid in water (A1) and methanol (B) at a flow rate of 0.25 mL/min. The gradient elution program was: 0–1 min, 10% B; 1–3 min, 10–33% B; 3–10 min, 33% B; 10–15 min, 33–50% B; 15–20 min, 50–90% B; 20–21 min, 90% B; 21–22 min, 90–10% B; 22–25 min, 10% B [24,25].

Mass spectrometric detection was performed using an AB Sciex QTRAP 6500+ system equipped with an electrospray ionization (ESI) source, capable of switching between positive and negative ion modes. For negative ion mode, the ion source temperature was 500 °C and spray voltage −4500 V; for positive ion mode, the ion source temperature was 550 °C and spray voltage +5500 V. Curtain gas was set at 30 psi, collision gas at 6 psi, and both ion source gas 1 and auxiliary gas at 50 psi.

Multiple reaction monitoring (MRM) was employed for quantitative analysis of 35 flavonoids. For each compound, the precursor ion (Q1), product ion (Q3), declustering potential (DP), entrance potential (EP), collision energy (CE), and collision cell exit potential (CXP) were individually optimized using standard solutions. Collision energies (CE) ranged from 15–45 eV to generate the most abundant and specific fragment ions. Retention times and ion information are listed in Table 1.

Quality control (QC) samples were prepared by pooling equal aliquots of all samples and injected every ten runs to monitor the stability of retention times, peak areas, and peak shapes throughout the analysis.

### 2.4. Data Preprocessing

First, Proteowizard software (v3.0.8789) was used to convert the obtained raw data into mzXML format (xcms input file format). Then, the XCMS package of R (v3.1.3) was used for peak identification, peak filtration, and peak alignment. The data matrix including the mass-to-charge ratio (*m*/*z*), retention time, and intensity was obtained. The precursor molecules in the positive and negative ion modes were obtained, and the data were exported to Excel for subsequent analysis.

### 2.5. Principal Component Analysis (PCA), Quality Assessment (QA), Quality Control (QC)

To screen reliable characteristic peaks for subsequent analysis, this study employed unsupervised principal component analysis (PCA) to independently model each sample group, thereby eliminating inter-group interference and identifying potential outliers (based on PC1 value distribution). After verifying data reliability through quality assurance (QA) procedures, quality control (QC) was further implemented. QC samples were prepared by mixing equal volumes of supernatant from the test samples and then combining them with standard solutions at a 1:1 volume ratio for instrumental analysis. QC samples were injected at regular intervals during each batch of sample analysis to monitor instrument stability. The relative standard deviation (RSD) of the peak areas of the standard in all QC samples was calculated to evaluate method reproducibility, with an RSD ≤ 15% considered acceptable. Finally, data were normalized to ensure comparability of metabolite levels.

### 2.6. Statistical Analysis of Metabolite Content

The statistical analysis of metabolite content in this study was based on standardized corrected data and then imported into the R language MetaboAnalystR package (v3.1.3) [26] for analysis. The imported data were then subjected to basic statistical analysis of metabolite content: identification and analysis of flavonoids, structural similarity analysis of flavonoid compound composition, differential analysis of flavonoids, screening of characterized flavonoids and correlation analysis of flavonoids in *P. amarus* leaves and shoots.

## 3. Results

### 3.1. Determination of Flavonoids by Liquid Chromatography–Tandem Mass Spectrometry (LC-MS/MS)

**Figure 1:**
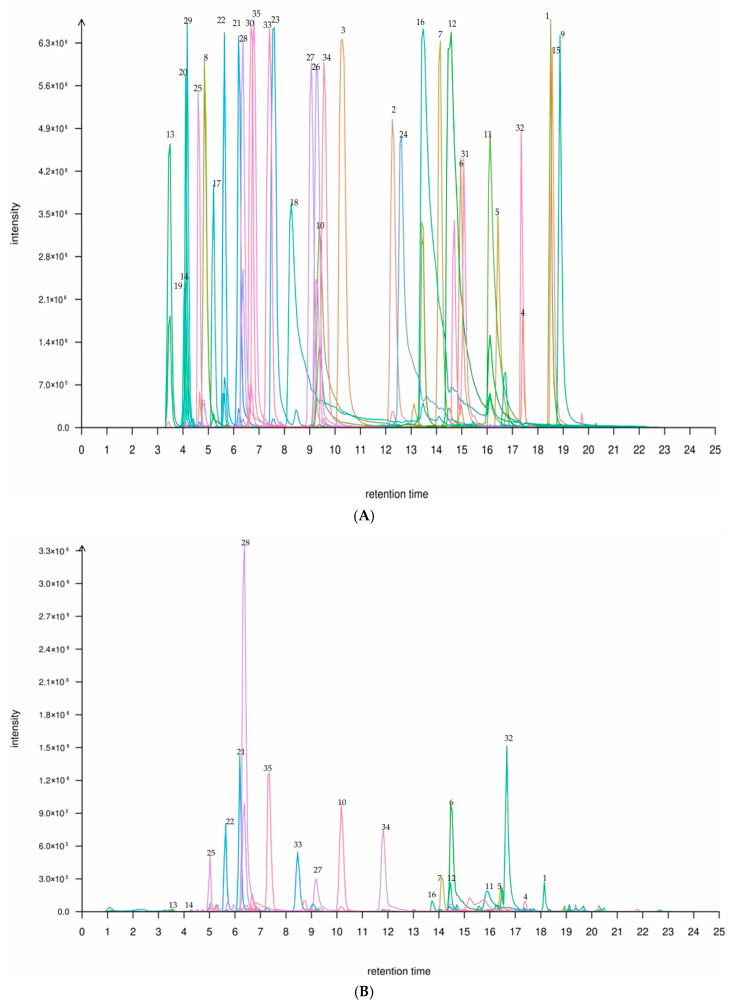

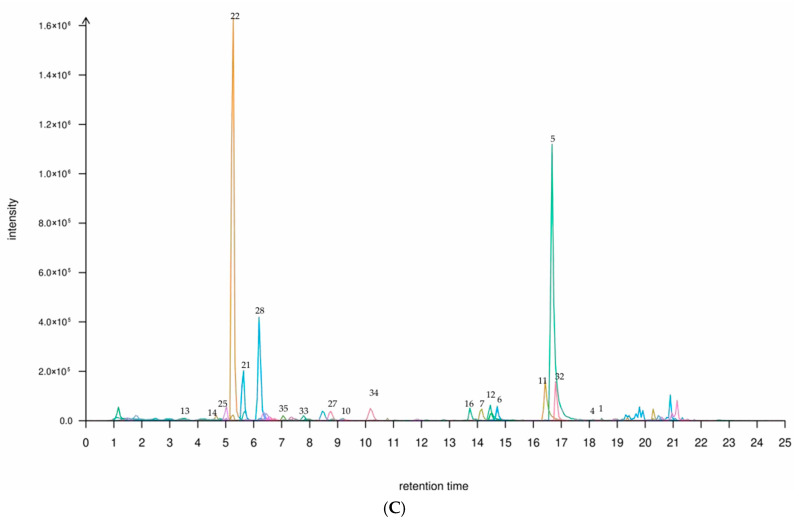
(**A**) A TIC plot of mixed labelling of 35 flavonoids in the leaf and shoot of *P. amarus*. (**B**) A TIC plot of 35 flavonoid compounds in the (**B**) leaf and (**C**) shoot of *P. amarus*. Each number corresponds to the serial number in Table 1.

Qualitative and quantitative analysis of flavonoid components in *P. amarus* leaves and shoots was performed using liquid chromatography–tandem mass spectrometry (LC-MS/MS). As shown in Figure 1, A presents the total ion chromatogram of mixed labeling of 35 flavonoid compounds in the leaves and shoots of *P. amarus*, while panels B and C provide the total ion chromatograms for the leaf and shoot samples, respectively. Target compounds were preliminarily identified by comparing retention times (RT) with those of reference standards. Subsequently, multiple reaction monitoring (MRM) was employed, where precursor ions were fragmented into product ions in the collision cell to reduce matrix interference, and structural inference was conducted using the product ion mode. The collision energy (CE) for each compound was individually optimized, ranging from 15 to 45 eV, to generate the most abundant and specific fragment ions. By measuring the response values (peak areas) of reference standards with known concentrations, a linear calibration curve (y = ax + b) between concentration and response was established for calculating the absolute content of target compounds in the samples.

Based on LC-MS/MS multiple reaction monitoring (MRM) quantification and fragment analysis, this study identified a total of 35 flavonoid compounds from *P. amarus* leaves and shoots. Table 1 provides detailed information for each compound, including compound name, characteristic fragment ions, retention time, standard curve parameters, and other relevant analytical data.

### 3.2. Results and Analyses of Principal Component Analysis (PCA), Quality Assessment (QA) and Quality Control (QC)

#### 3.2.1. PCA Results and Analyses

**Figure 2:**
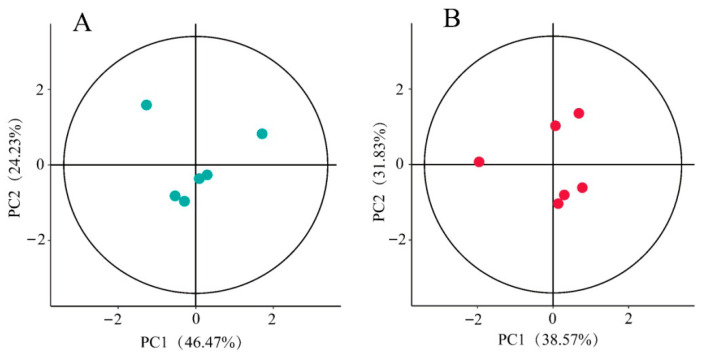
Plots of PCA scores of (**A**) leaf and (**B**) shoot samples of *P. amarus*, with each point in the figure representing a sample.

For quality assessment, unsupervised principal component analysis (PCA) was applied to the dataset comprising 6 leaf and 6 shoot samples (Figure 2). The Hotelling’s T2 multivariate test method was employed, followed by construction of 95% confidence interval ellipses. Samples plotting outside these ellipses would indicate poor data quality unsuitable for further metabolomic analysis. As shown in our results, all *P. amarus* leaf and shoot samples met quality criteria for subsequent analytical procedures.

#### 3.2.2. QA and QC Results and Analyses

The precision of the data and instrument performance were evaluated using relative standard deviation (RSD). An RSD value ≤ 15% demonstrates good method stability and reproducibility, indicating reliable data. As shown in Table 2, the RSD values for all flavonoid compounds were below 15%, confirming that the LC-MS/MS method for detecting 35 flavonoid classes in *P. amarus* leaves and shoots is stable and reliable. The resulting data are therefore sufficiently robust for subsequent analyses.

### 3.3. Statistical Analysis of Metabolite Content Results

#### 3.3.1. Identification and Analysis of Flavonoids in *P. amarus* Leaves and Shoots

**Figure 3:**
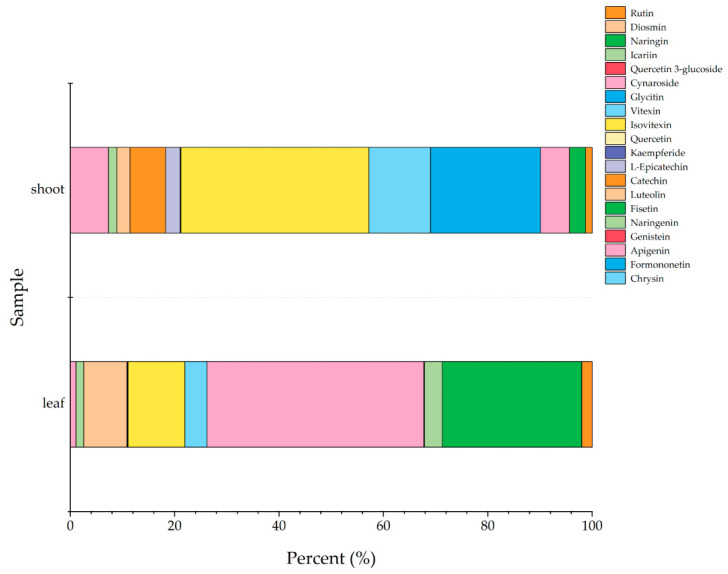
The average percentage content of the top 20 flavonoid compounds in leaf and shoot of *P. amarus* samples.

The percentage stacked bar chart is a data visualization method used to display the proportional composition of components within a whole. As shown in Figure 3, this study calculated the mean percentage content of 20 major flavonoid compounds in *P. amarus* leaf and shoot samples. The results were visualized using stacked bar charts to provide an intuitive comparison of flavonoid metabolite composition differences between the two sample groups. Among the 35 flavonoid compounds successfully detected by the LC-MS/MS method, 20 were selected for further comparative and visual analysis. These compounds were stably detected in all biological replicates, and their concentrations were above the limit of quantification, ensuring the reliability of the quantitative data. These 20 flavonoids were confirmed as the major forms present in *P. amarus* and served as the core focus for subsequent inter-tissue differential analysis.

The x-axis represents the percentage content of individual flavonoid compounds, while the y-axis indicates the samples, and the bar order corresponding to the flavonoid compounds from the top corresponds to the legend to the right. In the Supplementary Materials, the average flavonoid contents in the dry leaves and shoots of *P. amarus* are 0.19 and 0.02 mg/g, respectively. From the percentage stacked bar chart, it can be observed that cynaroside is the most abundant flavonoid in the leaves of *P. amarus*, while isovitexin shows the highest content in the shoots of *P. amarus*. Additionally, the leaves exhibited greater flavonoid diversity, containing unique compounds such as naringenin, luteolin, isovitexin, vitexin, cynaroside, naringin, rutin, and icariin. In contrast, the shoots contained distinct flavonoids not detected in the leaves, including catechin and glycitin.

#### 3.3.2. Structural Similarity Analysis of Flavonoid Compositions in *P. amarus* Leaves and Shoots

**Figure 4:**
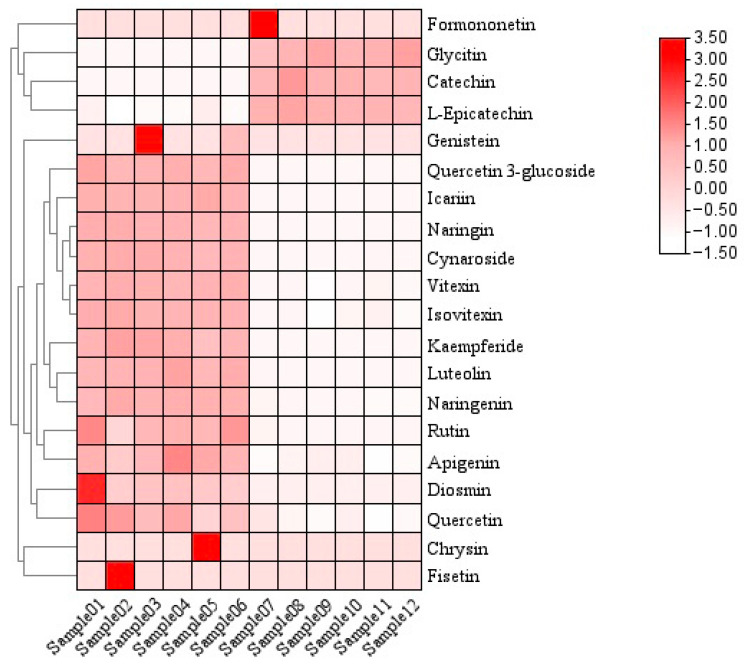
Heatmap clustering of structural similarity of flavonoids of leaves and shoots of *P. amarus*.

To investigate inter-sample similarities, cluster analysis was performed to construct sample clustering patterns, enabling systematic evaluation of similarities and differences among distinct samples or groups. As shown in Figure 4, heat map clustering was applied to the 20 most abundant flavonoids in *P. amarus*, with samples 1–6 derived from leaves and samples 7–12 from shoots.

**Figure 5:**
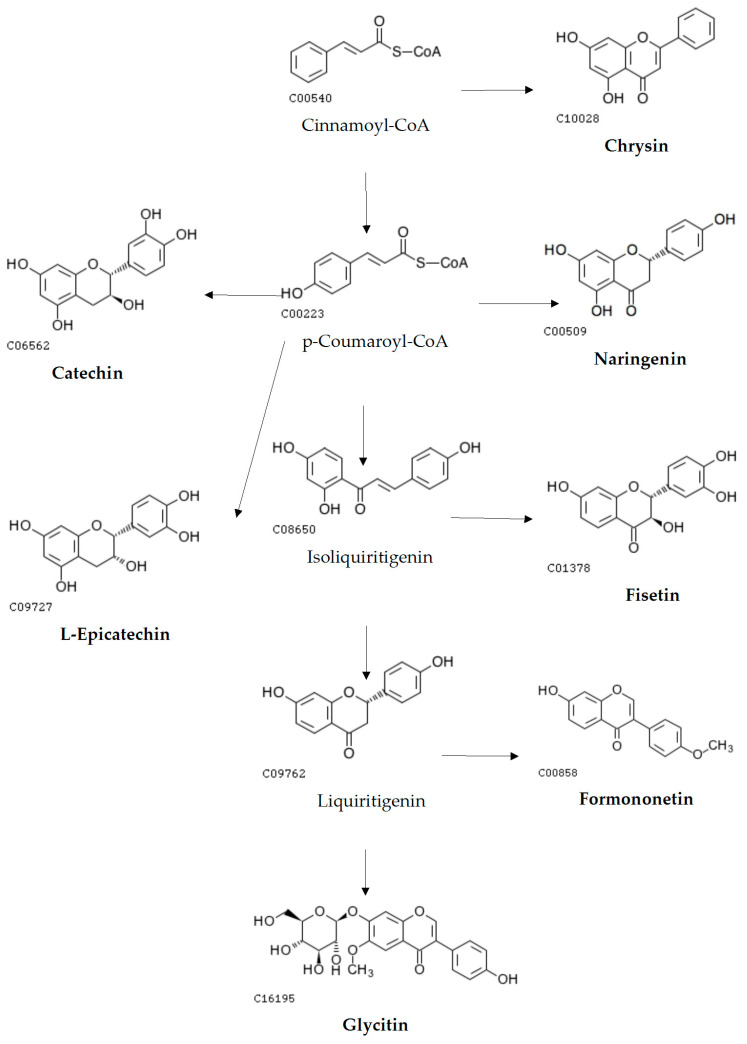
The main biosynthetic pathways of formononetin, L-epicatechin, glycitin, fistein and catechin. c06562, c00540, c10028 etc., represent the codes in the KEGG database.

**Figure 6:**
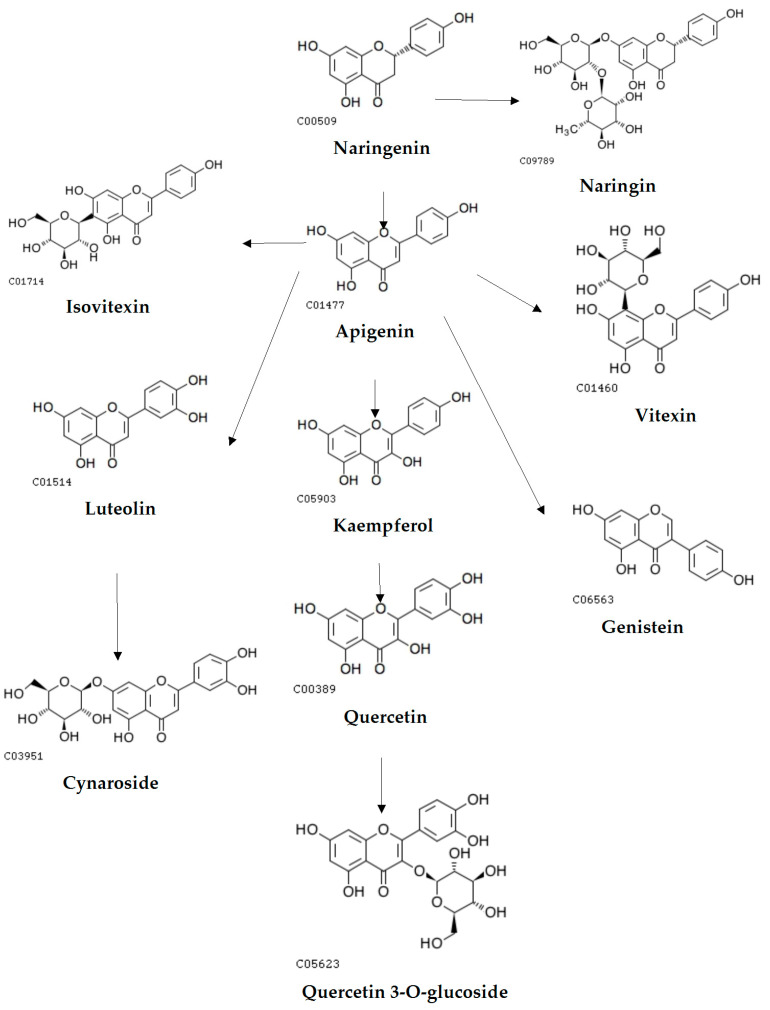
The biosynthetic pathways of the other group of flavonoids. c01514, c01477, c00509 etc., represent the codes in the KEGG database.

The vertical and horizontal axes represent the flavonoid compounds and the samples, respectively, including grouping information. The clustering tree on the left shows the similarity clustering of flavonoid compounds across the samples, the heatmap in the middle represents the content of flavonoid compounds, and the scale on the right shows the relationship between the colour and the content of flavonoid compounds. Positive values indicate positive correlations, negative values represent negative correlations, and darker colours signify stronger correlations. The clustering results indicate that these 20 flavonoid compounds are divided into two major groups. This is likely related to metabolic pathways, as shown in Figure 5 and Figure 6.

The two biosynthetic pathway diagrams reveal distinct biosynthetic routes between the two groups of flavonoids. One group, including formononetin, L-epicatechin, glycitin, and catechin, is primarily synthesized via the Cinnamoyl-CoA-derived pathway. In contrast, the biosynthesis of the other group predominantly centers around naringenin as a key intermediate.

#### 3.3.3. Differential Analysis of Flavonoids in *P. amarus* Leaves and Shoots

We employed Orthogonal Partial Least Squares Discriminant Analysis (OPLS-DA) to identify significantly differentiated flavonoid metabolites between *P. amarus* leaves and shoots. OPLS-DA is a supervised method requiring prior group information for analysis. This approach identifies discriminant factors (mathematically represented as weighted sums of all metabolites) that maximize inter-group separation. The method encodes discrete categorical variables into a continuous latent variable, enabling regression modeling between explanatory variables and this latent variable using least squares theory. As an advanced version of linear discriminant analysis, OPLS-DA is particularly suitable for omics data with substantial multicollinearity. The algorithm projects both predictor and response variables into a new orthogonal space (where dimensions are mutually independent and free from collinearity) to establish an optimal linear regression model. If the sample clusters from different groups are distributed in distinct regions, this indicates: The OPLS-DA model exhibits strong discriminatory power; Significant differences likely exist in metabolite profiles between the compared groups.

**Figure 7:**
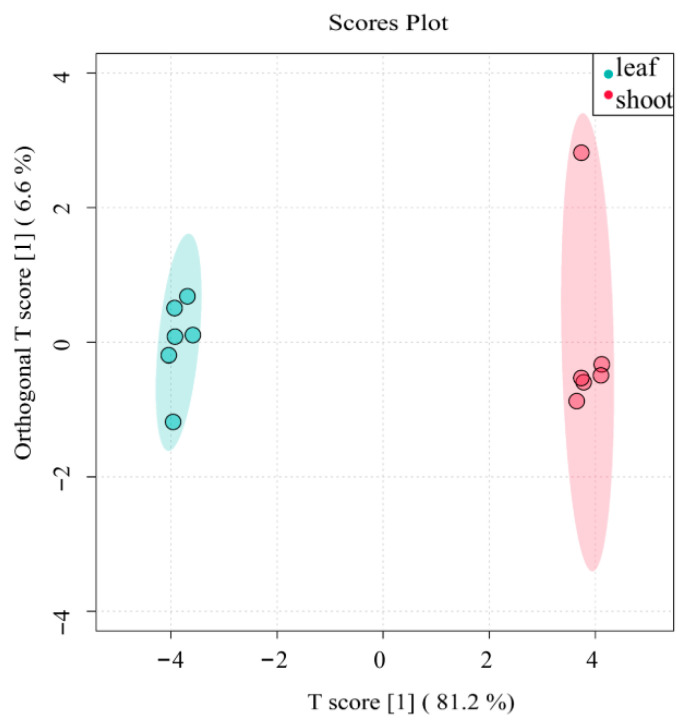
OPLS-DA score chart of flavonoids of *P. amarus* leaves and shoots. Each point corresponds to one sample, and the horizontal and vertical coordinates are the values of the two factors with the best discriminatory effect (score means the values of the two factors). The different colours represent different groupings, and the area marked by the ellipses is the 95% confidence region.

Interpretation of the S-plot in Figure 7 reveals that the highlighted data point in the right-most region corresponds to flavonoid compounds with high confidence and a strong contribution to the model’s predictive power. This clearly demonstrates a significant difference in flavonoid composition between *P. amarus* leaves and shoots.

#### 3.3.4. Screening of Characterized Flavonoids in *P. amarus* Leaves and Shoots

The Variable Importance in Projection (VIP) score, serving as a key metric in Orthogonal Partial Least Squares Discriminant Analysis (OPLS-DA), effectively identifies metabolites that contribute most significantly to metabolic differences between plant tissues or treatment groups. By calculating the weight of each metabolite in the model and its contribution to the classification model, metabolites with VIP scores (typically using a threshold > 1.0) are screened as key differential metabolites.

**Figure 8:**
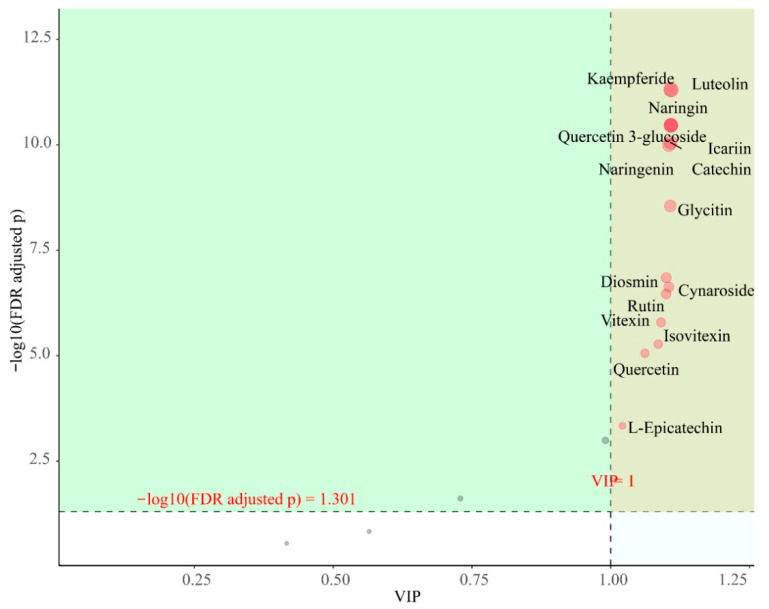
Importance diagram of flavonoids for OPLS-DA. Each point represents a flavonoid compound; the horizontal coordinate is the value of VIP and the vertical coordinate is the FDR-corrected *p*-value (log10 transformation).

As shown in Figure 8, when the screening conditions were VIP > 1 and *p* < 0.05, a total of 15 differential flavonoids were screened in the 12 samples, including kaempferide, diosmin, quercetin, quercitrin 3-glucoside, L-epicatechin, catechin, naringenin, rutin, glycitin, icariin, vitexin, isovitexin, luteolin, naringin, and cynaroside. These flavonoids have significant differences between subgroups and are also characteristic flavonoids that deserve our attention.

#### 3.3.5. Correlation Analysis of Flavonoids in *P. amarus* Leaves and Shoots

**Figure 9:**
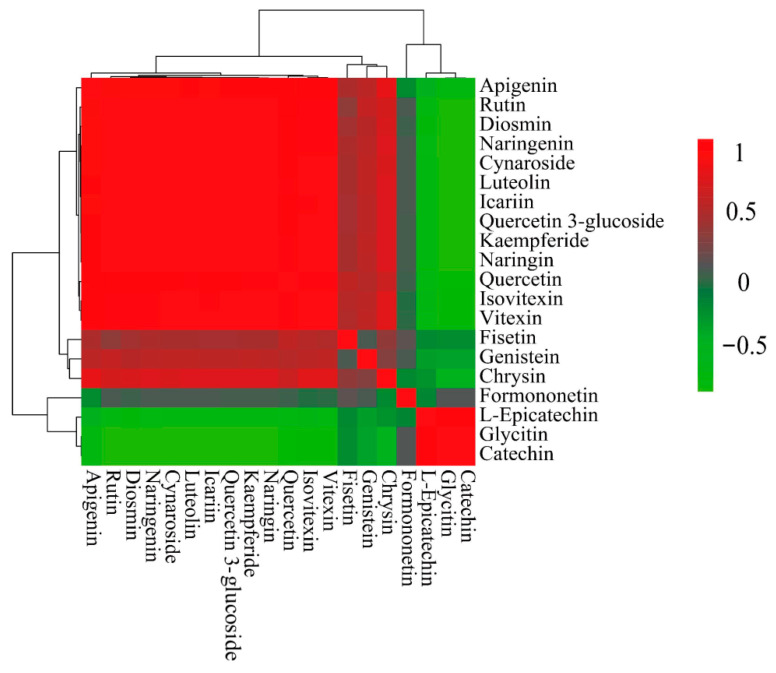
Correlation clustering of the top 20 flavonoids of the leaves and shoots of *P. amarus*. The correlation coefficients are indicated by colour, with positive correlations showing red and negative correlations showing green. Positive values indicate positive correlations, negative values represent negative correlations, and darker colours signify stronger correlations. The strength of the correlation is shown on the scale on the right.

The correlations of the top 20 flavonoids were further calculated, as shown in Figure 9, the correlation cluster diagram analysis shows that the 20 flavonoids are mainly divided into two groups. Formononetin, L-epicatechin, glycitin, and catechin are in the same branch, and the other 16 flavonoids are in the other branch, so they are divided into two groups. Formononetin, L-epicatechin, glycitin, and catechin are in one group, while chrysin, fisetin, genistin, kaempferide, diosmin, quercitrin 3-glucoside, quercetin, naringenin, apigenin, rutin, icariin, vitexin, isovitexin, quercitrin, cynaroside, and luteolin are divided into another group. These two groups of flavonoid compounds are negatively correlated between groups and positively correlated within groups.

## 4. Discussion

In this study, a targeted LC–MS/MS metabolomics approach was employed to systematically analyze and quantify flavonoid compounds in the leaves and shoots of *P. amarus*. The results showed good reproducibility among biological replicates. The optimized method exhibited low limits of detection and quantification, good linearity, and high sensitivity. Using this platform, a total of 35 flavonoid compounds were detected, among which 20 were quantitatively analyzed. This robust analytical platform provides a reliable tool for precisely comparing flavonoid accumulation in different tissues and reveals their distribution patterns more comprehensively than previous studies.

Principal component analysis (PCA) and OPLS-DA models indicated clear separation between leaf and shoot samples at the metabolic level, demonstrating significant tissue-specific metabolic differences. The number of flavonoid species detected in shoots was lower than that in leaves, suggesting that the missing flavonoids may be leaf-specific in *P. amarus* [27]. This pattern is consistent with metabolomic studies in other monocot plants, where flavonoids are typically more abundant in photosynthetic tissues and lower in storage or developing tissues. Similar results have been observed in rice, where roots exhibited the lowest flavonoid accumulation among different tissues [28]. Compared with previous studies on *P. amarus* flavonoids by Wang Hongbin et al. [10,11,12], this study newly identified 12 flavonoid compounds, including chrysin, formononetin, fisetin, genistein, diosmin, isoquercitrin, epicatechin, catechin, naringenin, glycitin, icariin, and naringin. Compared with closely related bamboo species [12], some common flavonoids were also detected in *P. amarus*, such as quercetin, luteolin, apigenin, rutin, vitexin, and isovitexin. However, orientin and isoorientin, commonly found in other related bamboo species, were not detected in *P. amarus*. These results indicate that the established LC–MS/MS method is highly efficient and specific, capable of capturing a broader flavonoid profile.

The differences in flavonoid abundance between leaves and shoots may reflect their distinct physiological functions and environmental adaptations. Leaves, directly exposed to sunlight, tend to accumulate flavonoids and flavonols such as luteolin and luteolin glycosides, which play crucial roles in photoprotection, antioxidant defense, and photosynthetic regulation. In contrast, shoots, located in low-light, high-humidity environments, contain unique metabolites such as catechin and glycitin, which may relate to tissue development and defense against soil pathogens. Environmental factors such as light intensity, temperature, and water availability are known to regulate the expression of key flavonoid biosynthesis genes [17,18,19,20,21], likely contributing to the observed tissue-specific differences.

From a bioactivity perspective, the higher levels of luteolin, luteolin glycosides, and vitexin in leaves suggest strong antioxidant and anti-inflammatory potential, consistent with their traditional medicinal use for “clearing heat, improving vision, and detoxification” [5]. Shoots, rich in catechin and glycitin, may have potential health-promoting effects, including lipid metabolism regulation and antimicrobial activity [8,9]. These tissue-specific metabolic features highlight complementary nutritional and pharmacological values of different plant parts, indicating that *P. amarus* could serve as a source of tissue-specific bioactive compounds [5,6,7]. Modern pharmacological studies also show that flavonoids confer diverse health benefits, including prevention of cardiovascular diseases, antitussive, antibacterial, hepatoprotective effects, and antioxidant activity through free radical scavenging [29]. Sun et al. [30] reported in a hyperlipidemic rat model that cynaroside and luteolin could improve lipid metabolism and mitigate hepatic steatosis. Tao et al. [31] found that vitexin and isovitexin could promote lifespan extension and health improvement in Caenorhabditis elegans by inhibiting insulin receptor activity, suggesting potential anti-aging and health-promoting effects. Stabrauskiene et al. [32] studied naringenin and naringin and revealed that they could suppress tumor growth and metastasis via multiple signaling pathways, indicating potential anticancer activity.

At the molecular level, this study found that the biosynthesis of formononetin, L-epicatechin, glycitin, fisetin, and catechin begins with stepwise condensation of p-coumaroyl-CoA and three molecules of malonyl-CoA, whereas other flavonoids are derived from naringenin via chalcone isomerase (CHI)-mediated cyclization. Previous studies have shown that flavonoid biosynthesis genes can be categorized into structural genes encoding enzymes and regulatory genes. Key enzymes involved in flavonoid biosynthesis include chalcone synthase (CHS), chalcone isomerase (CHI), isoflavone synthase (IFS), dihydroflavonol 4-reductase (DFR), and flavanone 3-hydroxylase (F3H) [33]. Fang Yumin et al. [34] noted that environmental factors such as light quality, temperature, and water availability significantly influence plant growth and secondary metabolite accumulation. Flavonoid biosynthesis is regulated by multiple factors, including transcription factors, endogenous non-coding small RNAs (miRNAs), and plant hormones. In Arabidopsis thaliana, MYB-domain transcription factors can individually regulate specific genes in the flavonoid biosynthetic pathway or cooperate with WD40 and bHLH proteins to form MYB–bHLH–WD40 (MBW) ternary complexes [35]. In the mybl2 mutant, loss of AtMYBL2 function leads to anthocyanin overaccumulation and upregulation of flavonoid biosynthesis-related structural and regulatory genes (DFR, LDOX, GL3, TT8, PAP1) [36,37]. In Arabidopsis, miR156 can influence flavonoid biosynthesis by regulating target SPL gene expression; increased miR156 activity promotes anthocyanin accumulation, whereas decreased activity increases flavonol levels [38]. Furthermore, studies have shown that targeted knockout of FtMYB45 via CRISPR/Cas9 in buckwheat significantly enhances the accumulation of multiple flavonoids [39].

Overall, the advanced LC–MS/MS method established in this study not only revealed the complex flavonoid metabolic features of *P. amarus* at both qualitative and quantitative levels but also provides new insights into plant physiological adaptation mechanisms. The method enables high-precision detection and profiling of tissue-specific flavonoid compositions, laying a solid analytical foundation for future pharmacological and nutritional studies of bamboo flavonoids.

## 5. Conclusions

In the analytical method developed for the 35 flavonoid compounds in this study, we ultimately selected 20 of them for in-depth analysis and presentation. This selection was based on the following criteria: these compounds were stably detected in all *P. amarus* plant samples and their concentrations were above the limit of quantification, ensuring the reliability of the quantitative data; they are the major flavonoid components in the plant tissues and exhibit significant distribution differences between leaves and shoots, which is crucial for revealing tissue-specific metabolite accumulation patterns. Focusing on these 20 flavonoids helps to present the core research findings more clearly.

In our study, flavonoids were investigated in six samples of *P. amarus* leaves and six samples of *P. amarus* shoots using targeted metabolomics based on LC-MS/MS, using 35 flavonoid standards to compare flavonoids in the leaves and shoots of *P. amarus*, and 12 additional flavonoids were found in these leaves and shoots in comparison to previous research. Significant differences in composition and structure were observed between these flavonoids, and 15 characterized differential metabolites were screened. There were significant differences in the flavonoid contents between the two different parts of the leaves and the shoots of *P. amarus*, and the contents of flavonoids in the leaves were higher than those in the shoots. Moreover, less flavonoids were distributed in the shoots than in the leaf parts. Our work is the first to use targeted metabolomics to analyze the flavonoids in two different parts of *P. amarus*, which lays a foundation for the subsequent directional separation and identification of flavonoids in *P. amarus*. The correlation cluster diagram analysis shows that the 20 flavonoids are mainly divided into two groups: catechin, L-epicatechin, formononetin, and glycitin in one, and the others divided into another group. Therefore, through the study of the flavonoid metabolism pathway, the reasons for dividing it into two groups were analyzed, which laid a foundation for further research on flavonoid metabolism in *P. amarus*. Thus, this study provides a scientific basis for further research on the different uses of different parts of the plant.

## Figures and Tables

**Table 1:** MRM detection parameters for 35 flavonoids in the leaves and shoots of *P. amarus*.

Number	Name	Precursor Ion (*m*/*z*)	Quantitative Product Ion (*m*/*z*)	DP (V)	EP (V)	CE (V)	CXP (V)
1	Chrysin	252.977	63.100	−105	−10	−52	−9
2	Daidzein	253.028	91.100	−98	−10	−52	−7
3	Liquiritigenin	255.102	119.100	−85	−10	−30	−1
4	Formononetin	266.985	252.100	−105	−10	−28	−5
5	Apigenin	268.898	117.100	−105	−10	−44	−1
6	Genistein	268.919	133.200	−105	−10	−42	−1
7	Naringenin	271.028	151.100	−75	−10	−26	−1
8	Glycitein	283.042	268.200	−95	−10	−26	−5
9	Biochanin A	283.065	268.100	−95	−10	−30	−5
10	Fisetin	284.890	134.800	−105	−10	−30	−7
11	Kaempferol	284.976	93.100	−115	−10	−54	−7
12	Luteolin	285.025	133.100	−100	−10	−44	−13
13	Catechin	289.012	245.100	−80	−10	−32	−5
14	L-Epicatechin	289.044	109.200	−95	−10	−36	−9
15	Kaempferide	299.010	284.000	−110	−10	−30	−5
16	Kaempferide	301.001	151.000	−95	−10	−30	−13
17	Taxifolin	302.997	285.100	−70	−10	−16	−5
18	Myricetin	16.872	150.800	−75	−10	−34	−9
19	Dihydromyricetin	318.957	192.700	−75	−10	−14	−3
20	Puerarin	415.101	295.200	−100	−10	−30	−5
21	lsovitexin	431.027	311.100	−95	−10	−30	−7
22	Vitexin	431.047	311.100	−105	−10	−30	−7
23	Genistin	431.076	268.100	−130	−10	−42	−5
24	Baicalin	445.151	269.200	−75	−10	−30	−5
25	Glycitin	445.162	283.100	−55	−10	−12	−5
26	Astragalin	447.089	284.000	−105	−10	−36	−5
27	Quercitrin	447.122	300.100	−95	−10	−36	−7
28	Cynaroside	447.127	285.000	−120	−10	−36	−5
29	Daidzin	461.104	253.100	−65	−10	−22	−5
30	Quercetin 3-glucoside	463.130	300.100	−120	−10	−36	−5
31	Silybin	481.107	125.100	−110	−10	−36	−11
32	Leariin	513.213	366.100	−160	−10	−38	−9
33	Naringin	579.260	271.200	−135	−10	−46	−5
34	Diosmin	607.260	299.100	−110	−10	−36	−7
35	Rutin	609.138	300.000	−165	−10	−52	−7

‘DP’ is Declustering Potential; ‘EP’ is Entrance Potential; ‘CE’ is Collision Energy; ‘CXP’ is Collision Cell Exit Potential.

**Table 2:** Retention time, linear regression equation, limit of quantification (LOQ), and repeatability (RSD%) of the target flavonoids.

Number	Flavonoids	Retention Time (min)	Linear Equation (Math.)	Correlation Coefficient (r)	Linear Range (ng/mL)	Limit of Quantification (LOQ) (ng/mL)	RSD (%)
1	Chrysin	18.57	y = 2.1 × 10^0.005^x + 1.04 × 10^4^	0.9919	0.2–100	0.2	2.95
2	Daidzein	12.27	y = 5.47 × 10^4^x + 5.37 × 10^3^	0.9958	0.8–400	0.8	1.39
3	Liquiritigenin	10.28	y = 1.74 × 10^5^x + 1.54 × 10^4^	0.9905	0.4–400	0.4	1.92
4	Formononetin	17.41	y = 2.43 × 10^5^x + 3.74 × 10^3^	0.9905	0.02–40	0.02	0.85
5	Apigenin	16.43	y = 1.35 × 10^5^x + 3.74 × 10^3^	0.9953	0.1–200	0.1	0.83
6	Genistein	14.96	y = 5.19 × 10^4^x − 238	0.9957	0.4–400	0.4	2.26
7	Naringenin	14.14	y = 1.72 × 10^5^x + 1.35 × 10^4^	0.9951	0.2–200	0.2	3.54
8	Glycitein	4.85	y = 6.6 × 10^5^x + 1.35 × 10^4^	0.9942	0.2–12.5	0.2	1.41
9	Biochanin A	18.51	y = 1.48 × 10^6^x + 7.88 × 10^3^	0.9922	0.02–20	0.02	2.50
10	Fisetin	9.40	y = 1.84 × 10^4^x − 5.83 × 10^3^	0.9907	0.8–4000	0.8	0.89
11	Kaempferol	16.13	y = 2.44 × 10^4^x + 4.96 × 10^3^	0.9943	2–2000	2	1.23
12	Luteolin	14.51	y = 1.23 × 10^5^x + 1.52 × 10^4^	0.9942	1–1000	1	3.68
13	Catechin	3.47	y = 2.99 × 10^3^x + 896	0.9945	2–4000	2	2.18
14	L-Epicatechin	4.11	y = 1.65 × 10^4^x + 6.04 × 10^3^	0.9960	2–500	2	1.98
15	Kaempferide	18.88	y = 2.2 × 10^5^x + 239	0.9940	0.1–200	0.1	4.63
16	Quercetin	13.48	y = 7.81 × 10^4^x − 1.31 × 10^4^	0.9945	1–2000	1	2.39
17	Taxifolin	5.20	y = 2.68 × 10^4^x + 3.25 × 10^3^	0.9950	0.4–800	0.4	1.01
18	Myricetin	8.28	y = 1.09 × 10^4^x − 6.81 × 10^4^	0.9910	10–10,000	10	1.96
19	Dihydromyricetin	4.05	y = 6.52 × 10^3^x + 0.419	0.9954	0.8–1600	0.8	1.84
20	Puerarin	4.17	y = 1.31 × 10^5^x + 2.01 × 10^3^	0.9929	0.2–100	0.2	1.67
21	lsovitexin	6.21	y = 6.46 × 10^4^x + 4.32 × 10^3^	0.9966	0.4–400	0.4	1.68
22	Vitexin	5.64	y = 9.91 × 10^4^x + 6.64 × 10^3^	0.9929	0.2–400	0.2	1.05
23	Genistin	7.56	y = 1.51 × 10^5^x + 1.39 × 10^4^	0.9914	0.4–400	0.4	2.85
24	Baicalin	12.60	y = 1.38 × 10^4^x − 2.29 × 10^3^	0.9947	2–10,000	2	4.13
25	Glycitin	4.83	y = 196x + 648	0.9923	20–5000	20	2.64
26	Astragalin	9.29	y = 6.62 × 10^4^x + 6.26 × 10^3^	0.9931	0.5–1000	0.5	3.51
27	Quercitrin	9.06	y = 5.18 × 10^4^x + 1.93 × 10^3^	0.9973	0.5–1000	0.5	3.41
28	Cynaroside	6.37	y = 6.72 × 10^4^x + 1.93 × 10^3^	0.9952	0.4–800	0.4	1.71
29	Daidzin	4.61	y = 3.29 × 10^4^x + 885	0.9909	0.5–1000	0.5	1.80
30	Quercetin 3-glucoside	6.80	y = 7.11 × 10^4^x + 7.24 × 10^3^	0.9917	0.5–1000	0.5	1.91
31	Silybin	15.08	y = 5.58 × 10^4^x + 5.04 × 10^3^	0.9915	0.5–1000	0.5	1.46
32	Icariin	17.35	y = 1.67 × 10^4^x + 1.74 × 10^3^	0.9930	1–1000	1	1.32
33	Naringin	7.42	y = 3.58 × 10^4^x + 7.59 × 10^3^	0.9915	1–2000	1	2.36
34	Diosmin	9.57	y = 7.99 × 10^4^x + 6.10 × 10^3^	0.9916	0.4–800	0.4	2.56
35	Rutin	6.69	y = 3.90 × 10^4^x + 3.55 × 10^3^	0.9965	1–1000	1	2.30

‘RSD’ is relative standard deviation.

## Data Availability

The original contributions presented in this study are included in the article/Supplementary Material. Further inquiries can be directed to the corresponding author.

## Supplementary Materials

The following supporting information can be downloaded at https://www.mdpi.com/article/10.3390/metabo15110709/s1.
